# Probing multivalency in ligand–receptor-mediated adhesion of soft, biomimetic interfaces

**DOI:** 10.3762/bjoc.11.82

**Published:** 2015-05-12

**Authors:** Stephan Schmidt, Hanqing Wang, Daniel Pussak, Simone Mosca, Laura Hartmann

**Affiliations:** 1 Universität Leipzig, Institut für Biochemie, Johannisalle 21–23, D-04103 Leipzig, Germany; 2 Max Planck Institute of Colloids and Interfaces, Research Campus Golm, 14424 Potsdam, Germany; 3 Heinrich-Heine-Universität Düsseldorf, Institut für Organische und Makromolekulare Chemie, Universitätsstr. 1, 40225 Düsseldorf, Germany

**Keywords:** bio-interfaces, cell mimetic, glycocalyx, glycopolymer, molecular recognition, RICM, specific adhesion

## Abstract

Many biological functions at cell level are mediated by the glycocalyx, a dense carbohydrate-presenting layer. In this layer specific interactions between carbohydrate ligands and protein receptors are formed to control cell–cell recognition, cell adhesion and related processes. The aim of this work is to shed light on the principles of complex formation between surface anchored carbohydrates and receptor surfaces by measuring the specific adhesion between surface bound mannose on a concanavalin A (ConA) layer via poly(ethylene glycol)-(PEG)-based soft colloidal probes (SCPs). Special emphasis is on the dependence of multivalent presentation and density of carbohydrate units on specific adhesion. Consequently, we first present a synthetic strategy that allows for controlled density variation of functional groups on the PEG scaffold using unsaturated carboxylic acids (crotonic acid, acrylic acid, methacrylic acid) as grafting units for mannose conjugation. We showed by a range of analytic techniques (ATR–FTIR, Raman microscopy, zeta potential and titration) that this synthetic strategy allows for straightforward variation in grafting density and grafting length enabling the controlled presentation of mannose units on the PEG network. Finally we determined the specific adhesion of PEG-network-conjugated mannose units on ConA surfaces as a function of density and grafting type. Remarkably, the results indicated the absence of a molecular-level enhancement of mannose/ConA interaction due to chelate- or subsite-binding. The results seem to support the fact that weak carbohydrate interactions at mechanically flexible interfaces hardly undergo multivalent binding but are simply mediated by the high number of ligand–receptor interactions.

## Introduction

Vast amounts of biological processes are mediated by interactions between membrane proteins and carbohydrates of the glycocalyx, a glycan coating enveloping prokaryotic or eukaryotic cells. By specific binding to cell receptors, the carbohydrate units of the glycocalyx control important processes such as cell adhesion, communication and inflammatory response [1]. For this reason great effort is set forth to identify the key principles of carbohydrate/protein receptor interaction and to utilize carbohydrate structures as drugs, e.g., in cancer treatment or pathogen-related diseases [2–3]. A well-established key principle of carbohydrate–receptor interactions is multivalency. Natural carbohydrate ligands are typically oligomers consisting of multiple subunits of varying complexity. When binding to receptors this leads to a receptor clustering or so-called glycocluster effect. However, multivalency even goes further: For example when cells form contact layers of surface anchored carbohydrates the glycocalyx interacts also with surface anchored membrane receptors. Thus, the interactions between these surfaces are again multivalent interactions, just on a larger scale between two surfaces. Such carbohydrate based multivalent surface–surface interactions represent a large range of crucial biological events such as initial cell adhesion processes or pathogen invasion in host tissue. Nevertheless, ligand–receptor interactions are typically characterized by studying the binding affinity of freely dissolved ligands without surface anchorage. Typical assays in this context are “chip”-based methods like surface plasmon resonance, quartz crystal microbalance or impedance spectroscopy where the affinity of ligands is measured against only one surface, the biochip surface [4–7]. In addition, other typical affinity assays like micro-calorimetry or the agglutination do not take the effect of multivalent surfaces into account. However, merely studying affinity of dissolved ligands does not always capture the biological situation, e.g., when a layer of surface anchored carbohydrates of the glycocalyx interacts with also surface anchored membrane receptors.

**Figure 1:**
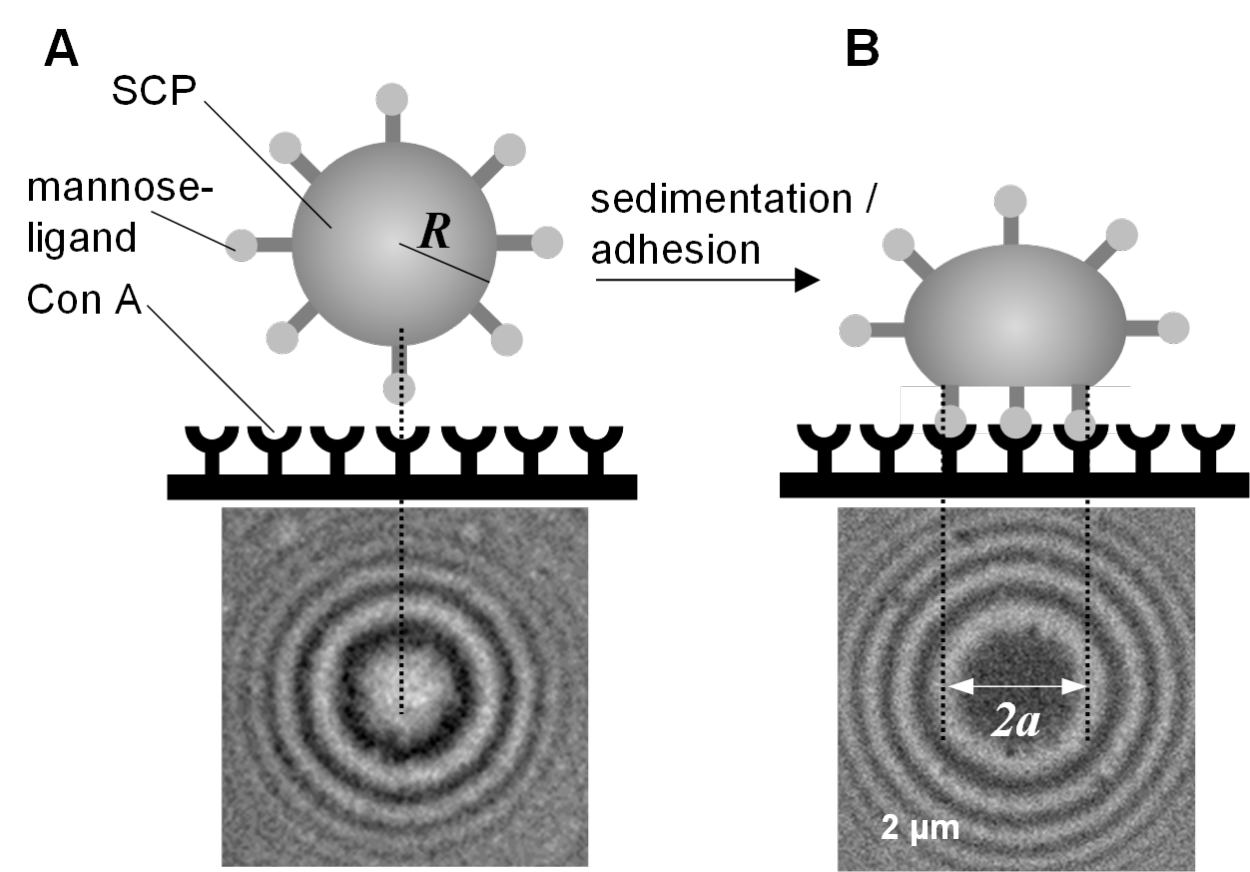
SCP adhesion measurement sketch (top): A mannose-functionalized PEG-SCP sediments onto a Concanavalin A (ConA) receptor surface (left), then mannose units bind to ConA inducing adhesion and mechanical deformation of the SCP (right). The contact area of the SCP can be read out via reflection interference contrast microscopy (RICM) from the central circular interference minimum (bottom).

In order to directly study the interaction of surface anchored carbohydrate ligands with a receptor surface, we developed a new method using carbohydrate coated hydrogel particles, also called soft colloidal probes (SCPs) that undergo mechanical deformation when coming into contact a with receptor surface [8–11]. The mechanical deformation is a measure of the sum over all specific interactions between the carbohydrate ligand and the protein receptor layer. Detection of the interaction energy is straight forward using the contact area of the SCP with the protein layer via reflection interference contrast microscopy (RICM) (Figure 1). The contact area can be related to the specific adhesion energy *W_adh_* of the SCP adhering to a surface using the JKR Model:

[1]
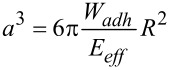


where *a* is the radius of contact, *R* is the radius of the SCP and *E_eff_* = [4*E*/3(1−*ν*^2^)] its effective elastic modulus, with *ν* the Poisson ratio and *E* the Young's modulus of the SCP.

Importantly, the SCP and the receptor surface represent a reduced cell-matrix model system that roughly mimics the biological context and thus allows studying the effect of various parameters affecting the interaction between surface anchored binding partners, e.g., the mechanical flexibility of the interfaces, the surface presentation and density of the binding partners. In this work, we focus on the latter aspects. More specifically, we study the effect of linker type and ligand density on the interaction between sugar-ligands and receptors on a surface. It is well known that multivalency and linker type can drastically affect the interaction on a single molecule level [12]. Since interactions between surfaces are multivalent per se it is important to also study the effect of parameters that may affect this “surface multivalency” and the resulting interaction strength.

In the previous studies, we looked at the interactions between the ConA receptor and its natural sugar ligand mannose. The receptor was immobilized on a glass coverslip and mannose ligands were coupled on the SCPs. Attachment of the sugar ligands on the SCP was achieved by coupling of amine-functionalized mannose to carboxy-functionalized SCPs. Carboxy groups on the SCP were introduced by a radical grafting process where incubation of the PEG microgels with benzophenone and acrylic acid lead to poly(acrylic acid) grafts on the PEG backbone.

Using this model system, we could already show that the mechanical flexibility of the interface presenting the sugar ligands has a pronounced effect on the resulting adhesion energy [8]. This was shown by varying the length of poly(ethylene glycol) chains that establish the soft hydrogel matrix of the SCPs and measuring the interactions between mannose SCPs and ConA surfaces.

In this work, we aim to control the sugar ligand concentration, density and linking chemistry on the SCPs by adopting the radical grafting process for different acrylic monomers. We hypothesize that the use of acrylic acid (AA), methacrylic acid (MA) and crotonic acid (CA), respectively, will lead to different grafting length and grafting density. For example, MA has a much higher reactivity compared to AA and CA. This should lead to long and dense MA grafting to the PEG SCPs. The reactivity of AA is comparatively lower thus increasing the probability for chain transfer reactions and shorter grafts [13]. CA cannot be homopolymerized through free radical polymerization, meaning that only single CA units will be attached to the PEG chains [14]. However, other phenomena may also affect the resulting grafting density such as different tendency of grafting from and grafting to between the different monomers. Thus, in the first part, we study the functionalization degree of the PEG-SCPs for MA, AA and CA grafts via titration and zeta potential measurements. These systems will then be functionalized with mannose ligands to obtain PEG-SCPs with varying ligand density. In the second part, we study the adhesion energy of the functionalized SCPs on ConA receptor-functionalized glass slides. Depending on the type of grafting process and the concentration of functional groups attached to the SCP, the differences in adhesion energy due to surface presentation and density of mannose ligands are discussed.

## Results and Discussion

### PEG-microgel synthesis and carboxylic acid grafting

We started out synthesizing carboxylic acid-functionalized PEG particles with varying functionalization degree by grafting three types of carboxylic acid monomers to the PEG network: methacrylic acid (MA), acrylic acid (AA) and crotonic acid (CA). Special emphasis is on the precise control of the functionalization degree that ultimately controls the sugar ligand group concentration for adhesion energy measurements. Therefore, the carboxylic acid-functionalized particles were analyzed by titration with toluidine blue O (TBO) and zeta potential measurements. In addition, we explore the effect of the reaction conditions, such as concentration of reagents or UV irradiation time on the functionalization degree of CA-functionalized particles.

#### Comparison of the functionalization of PEG SCPs with different carboxylic acid monomers

**Scheme 1:**
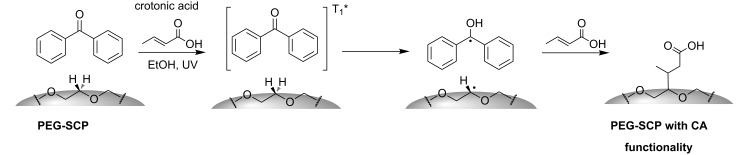
PEG functionalization is based on radical benzophenone photochemistry and subsequent addition of carboxylic monomers (CA is depicted as an example). In the first step, benzophenone abstracts hydrogen from the polymer surface to generate surface radicals. In the presence of α,β-unsaturated carboxylic acids, the macroradical initiates grafting via the radical-polymerization mechanism.

Recently, we introduced micrometer-sized SCPs composed of crosslinked PEG-diacrylamide hydrogels as probes [15]. Here, we used the ability of PEG-diacrylamide to phase separate from an aqueous solution to form microscopic droplets by means of precipitation using sodium sulfate as a kosmotrope. At concentrations of 5 mg/mL PEG-diacrylamide and 1 M sodium sulfate, the polymer–water interactions are less favorable than polymer–polymer and water–water interactions, thus polymer droplets form which are then UV polymerized. Using neutral and inert PEG scaffolds for studying specific adhesion is advantageous because it reduces nonspecific interactions and also complicates the bioconjugation. To deal with this problem, we adjusted a surface chemistry route including radical generation at the PEG backbone by UV irradiation [15]. This enabled the addition of unsaturated carboxylic moieties onto the PEG scaffold (Scheme 1).

It is known that benzophenone can absorb the energy of photons to excite the electron in its carbonyl group from ground state (S0) to the excited state (S1) or (S2) depending on the wavelength used. Subsequently, the excited electron can go to the triple state (T1) through intersystem crossing (ISC). At the T1 state, the benzophenone molecule has an excited electron which is highly reactive, and can abstract a hydrogen atom from the PEG backbone. The abstracted hydrogen atom generates a radical on the PEG-backbone and a semipinacol radical on the benzophenone carbonyl group [16]. In the presence of α,β-unsaturated carboxylic acids, the PEG-backbone radical attacks the unsaturated carbon bond resulting in the grafting of the carboxylic acid on the PEG-SCPs.

**Figure 2:**
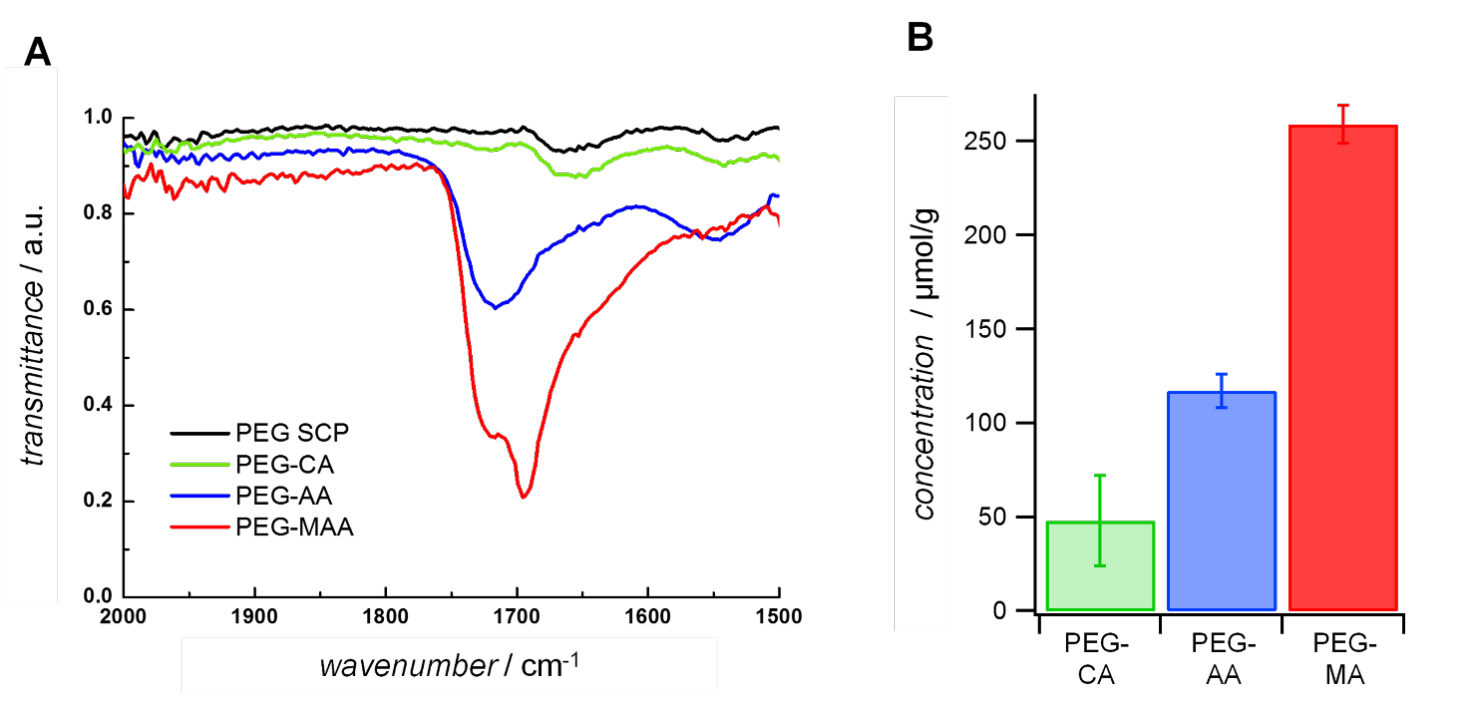
A) ATR–FTIR spectroscopy signifying carbonyl group at around 1720 cm^−1^ and successful grafting; B) Results of the TBO titration of the grafting of carboxylic acid onto PEG SCPs.

The grafting density of MA, AA and CA on PEG-SCPs was studied under the same reaction conditions for the different carboxylic acids. As a first test, the presence of carboxylic acid groups in the SCPs was evaluated by ATR–FTIR (Figure 2A). An increase of the peak around 1720 cm^−1^ can be observed that corresponds to the signal of the carbonyl group of the carboxylic acid molecule, showing the successful grafting of the acid molecules and that the carbonyl density increases from CA, AA to MA. In order to quantify the carboxylic acid group concentration, a colorimetric titration was carried out using TBO. As expected, the CA grafting resulted in the lowest density, AA grafting intermediate density, and MA grafting in largest density of carboxylic acid groups. The overall ratio for CA, AA and MA was 1:2.4:5.4. (Figure 2B). Zeta potential measurements confirmed this trend (see Supporting Information File 1, S1). It is important to note that not only the surface of the SCPs is functionalized with carboxlic acid groups, but the whole bulk of the particles. This was confirmed via confocal Raman microscopy indicating a homogeneous distribution of functional groups (Supporting Information File 1, S2).

The observed increase in grafting density of MA over AA is well established in the literature [13,17–18]. For example, Yang et al. [17] compared the grafting density of MA and AA on low density polyethylene (LDPE) films through measuring the weight increase of the film. With the same reaction conditions and reaction time, the weight increase of methacrylic grafted film was 2 to 5-folds heavier than acrylic acid grafted films, which is in good agreement with our results. The increased grafting reactivity of MA can be explained by the substituent on the double bond. The methyl group of MA may activate the double bond due to hyperconjugation. In addition, the activation energies for polymerization of MA is lower than for AA [19], leading to longer MA grafting units compared to AA. Another interesting fact is that the kinetics of polymerization initiation is slower compared to propagation in case of AA units whereas the opposite is the case for MA. This behavior further increases the length of MA grafts compared to AA. Furthermore, we assume that MA undergoes a strict ‘grafting from’ mechanism, whereas for AA there is the possibility of a ‘grafting onto’ mechanism. This is because we observed AA homopolymers in the reaction mixture after particle functionalization as measured via HPLC, whereas no such MA polymers were found (data not shown). It appears that such free poly(acrylic acid) species could attach via a grafting onto mechanism. On the other hand, due to the conformation of the polymer in solution and the steric hindrance, the grafting density should be lower for grafting onto in comparison to the grafting from.

In case of CA it is generally believed that it cannot be homo polymerized via free radical polymerization [14]. CA contains a 1.2-disubstituted ethylene exhibiting high steric hindrance, which might explain the low reactivity compared to the MA and AA. Therefore, radicals at the β-carbon may not be able to further react with other monomer molecules leaving only one CA molecule per graft of the PEG backbone. Since the overall reaction conditions were kept constant for MA, AA and CA grafting, it is clear that CA shows the lowest grafting density due to the expected inability to homo polymerize.

#### Influence of the reaction conditions for crotonic acid grafting

As CA forms just one carboxy group per radical on the PEG-SCPs, the CA functionalization procedure should give the best control over the actual number of attached functional groups. In order to control the density of CA grafting, several parameters such as, monomer concentration, initiator concentration, irradiation time and reaction conditions, were taken into account. Our intermediate goal was to maximize the CA concentration on the PEG backbone. Therefore, we increased the amount of CA from 1.7 mol/L to 3.5 mol/L (solubility maximum), while the concentration of the other reactants staid constant. As shown by TBO titration, the grafting density on the order of 52 ± 5 µmol/g was invariant to the CA reaction concentration (see Table 1). This suggests that under the applied CA concentration range the grafting density might be limited by the radicals formed on the PEG network. The amount of radicals formed on the PEG chain is not affected by the presence of CA, but rather by the benzophenone concentration which was kept constant so far. Therefore, we varied the benzophenone concentration from 60 mmol/L to 420 mmol/L. From the results shown in Table 1, it can be seen that the benzophenone concentration indeed affected the final functionalization degree. Overall, an increase by 50% was observed when comparing reactions with the lowest and highest amount of benzophenone. Therefore, CA grafting increases with increasing benzophenone concentration, confirming that benzophenone concentration affects the formation of surface radicals, which also suggests a grafting from mechanism for CA.

**Table 1:** Variation of the crotonic acid and benzophenone concentration and its influence on the grafting. The functionalization degree increases with increasing benzophenone concentration regardless of the crotonic acid concentration.

CA concentration (mol/L)	Benzophenone concentration (mmol/L)	Functionalization degree (µmol/g)

1.7	60	36 ± 2
	140	48 ± 10
	420	57 ± 5
3.5	60	37 ± 2
	140	52 ± 5
	420	60 ± 3

**Figure 3:**
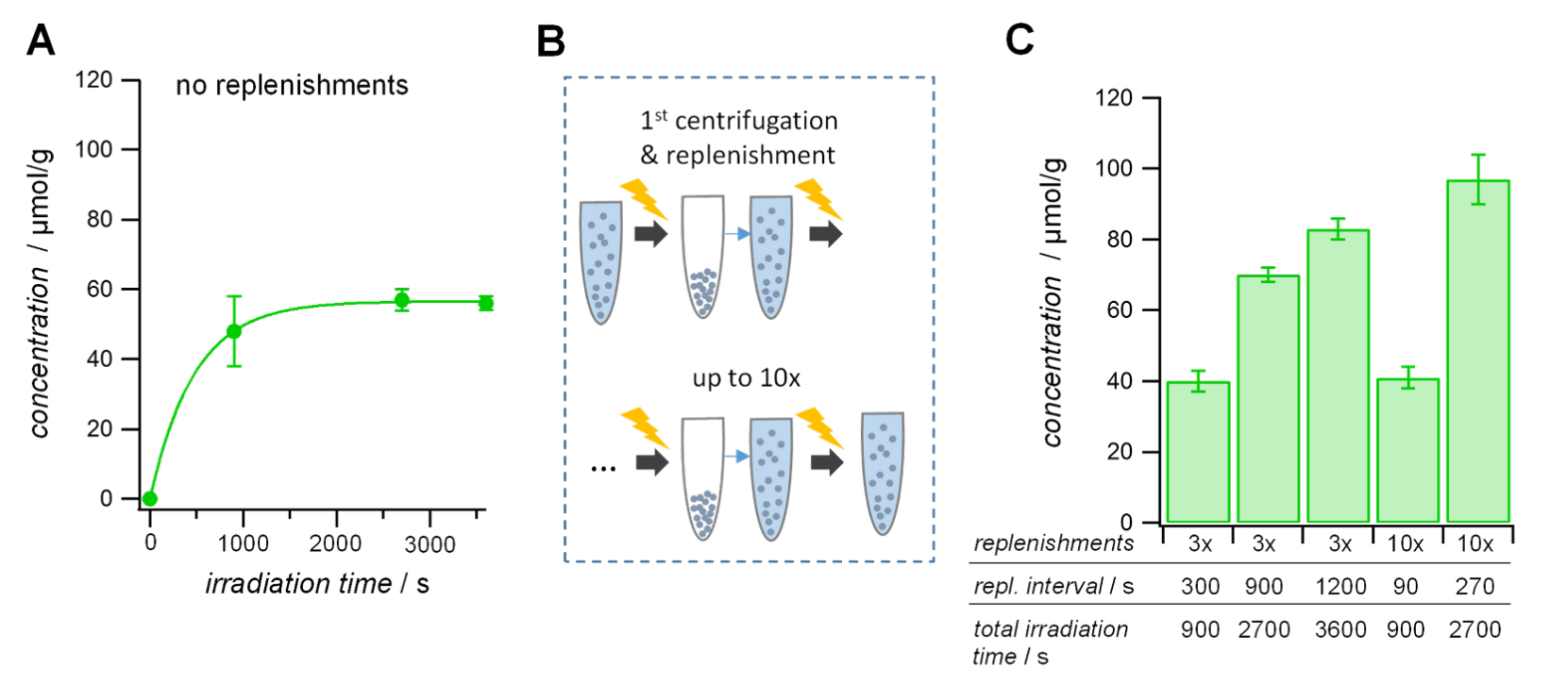
A) CA functionalization degree as a function of the irradiation time. The solid line represents an exponential fit in indicating the effective time constant of CA grafting. B) Graphical scheme of the reactants replenishing procedure. The solution of benzophenone and CA was replaced with a fresh solution after different irradiation intervals up to ten times. C) Results of the solution exchange procedure. By refreshing the solution the functionalization degree increases.

The benzophenone concentration has an effect on the CA functionalization degree but the tunable range is still narrow. Therefore, we varied the irradiation time as this might lead to a more sustained grafting process. We varied the irradiation time (900 s, 2700 s and 3600 s) at concentrations of 140 mM benzophenone and 1.7 M CA and determined the functionalization degree by TBO titration. We found a rather modest increase by 14% with longer irradiation times from 900 s to 3600 s, respectively. From this result we conclude that at an irradiation time of 2700 s, the reaction is completed and further irradiation does not further increase the functionalization degree (Figure 3A). This suggests that at this point benzophenone was completely consumed in form of benzopinacol and no further grafting could occur. To improve the availability of benzophenone in the reaction mixture we replenished the reaction solution with new reactants, i.e., benzophenone and CA at 140 mM and 1.7 M, respectively (Figure 3B). After the PEG-SCPs were irradiated, the reaction solution was exchanged with fresh reactants via centrifugation, decantation and re-dispersion of the PEG-SCPs before the next irradiation step. Using this approach, we varied the number of replenishing steps as well as irradiation interval between the replenishing step and measured the resulting functionalization degree (Figure 3C).

From the results in Figure 3C, it can be seen that with replenishing the solution, the CA concentration on SCPs was improved significantly. Especially, when the solution was exchanged at intervals of 200–900 s the grafting was rather efficient. For smaller intervals (e.g., 90 s) there is still enough non-reacted benzophenone so that even ten times replenishing did not show a significant increase over non-replenished solution that was irradiated for the same time. The grafting becomes rather inefficient if the replenishing interval is larger than 900 s because then most of the benzophenone has been already consumed before the replenishing step. This can be seen from Figure 3A showing the functionalization degree of PEG particles as a function of time. At around 900 s the exponential fit begins to level off significantly indicating that most of the benzophenone has been consumed already. Therefore replenishing at 1200 s intervals proved to be less efficient as compared to 270 s intervals (Figure 3C). Overall, we found that replenishing at about 200–600 s was the best option in order to increase the CA functionalization degree and to keep both the reaction time and number of replenishing steps in a reasonable range. Using this procedure, we achieved carboxylic acid functionalization degrees similar to AA or MA. This allows for comparative SCP binding studies on the type of grafts introduced to the PEG network.

### Adhesion studies with mannose-functionalized PEG-SCPs

#### Functionalization of carboxylate-functionalized PEG-SCPs with mannose

To study the influence of the degree of functionalization and the grafting type on particle adhesion, we prepared five different SCP systems functionalized with different concentration of carboxylic acid groups: PEG-MA, PEG-AA, PEG-CA_low_, PEG-CA_middle_, PEG-CA_high_. PEG-MA and PEG-AA are SCPs having a polyacid chain on the backbone, whereas the tree different PEG-CA SCPs present single acid moieties attached to the PEG network. As shown in the previous chapter, the functionalization degree of CA was tuned by varying the reaction conditions. The resulting concentrations of carboxylic acids in the PEG network are detailed in Table 2.

**Table 2:** Results of the functionalization of the carboxylate-functionalized particles with mannose.

PEG-SCP type	Concentration of carboxyl groups (µmol/g)	Concentration of mannose groups (µmol/g)

PEG-CA_low_	36 ± 2	22 ± 6
PEG-CA_middle_	57 ± 5	44 ± 8
PEG-CA_high_	97 ± 7	89 ± 9
PEG-AA	117 ± 9	56 ± 14
PEG-MA	259 ± 24	193 ± 29

In order to study the specific interactions between the mannose ligands and ConA receptors, we used aminoethyl-linked mannose and coupled it via standard coupling chemistry on the PEG SCPs [8] (Supporting Information File 1, S5). The number of mannose functionalities and therefore the degree of functionalization can be directly measured via UV titration with TBO of the unreacted carboxy groups and compared to the pure carboxylate-functionalized probes (Supporting Information File 1, S3). The coupling of mannose to carboxylic acids was not quantitative, not all carboxylic acid would couple to mannose units. However, controlled variation of the mannose group concentration on SCPs could still be achieved due to the significant decrease of the carboxylic acid groups after coupling revealing the number of attached mannose ligands. The controlled variation of mannose units will be used to further investigate the adhesion behavior in the following section.

#### Determination of specific adhesion via SCP as function of mannose ligand density

For the SCP adhesion measurements, we used ConA-functionalized glass coverslips. We expect a dense packing of the receptor protein on the glass coverslips using a covalent attachment protocol as described earlier [15]. In a typical binding assay, the SCPs were dispersed in buffer and sediment onto the receptor surface (Figure 1). Upon contact with the ConA receptors, ligands and receptors bind and the SCPs adhere to the receptor surface. Since they are soft, they formed a distinct contact area which can be evaluated using the JRK approach to calculate the adhesion energy *W*, see Equation 1 (Supporting Information File 1, S6). Qualitatively speaking, the larger the contact area between the mannose presenting SCPs and ConA surface the larger the adhesion energy. When measuring specific adhesion between ligands and receptors, it is essential to carry out control experiments to ensure that the adhesion is indeed due to specific interactions. Therefore, as control an inhibition experiment was performed by adding α-methyl-D-mannose as low molecular weight inhibitor blocking the binding sites of ConA. As a result, all probes detached from the surface and do not adhere anymore indicating that the interaction between SCPs and ConA surface was specific [15].

**Figure 4:**
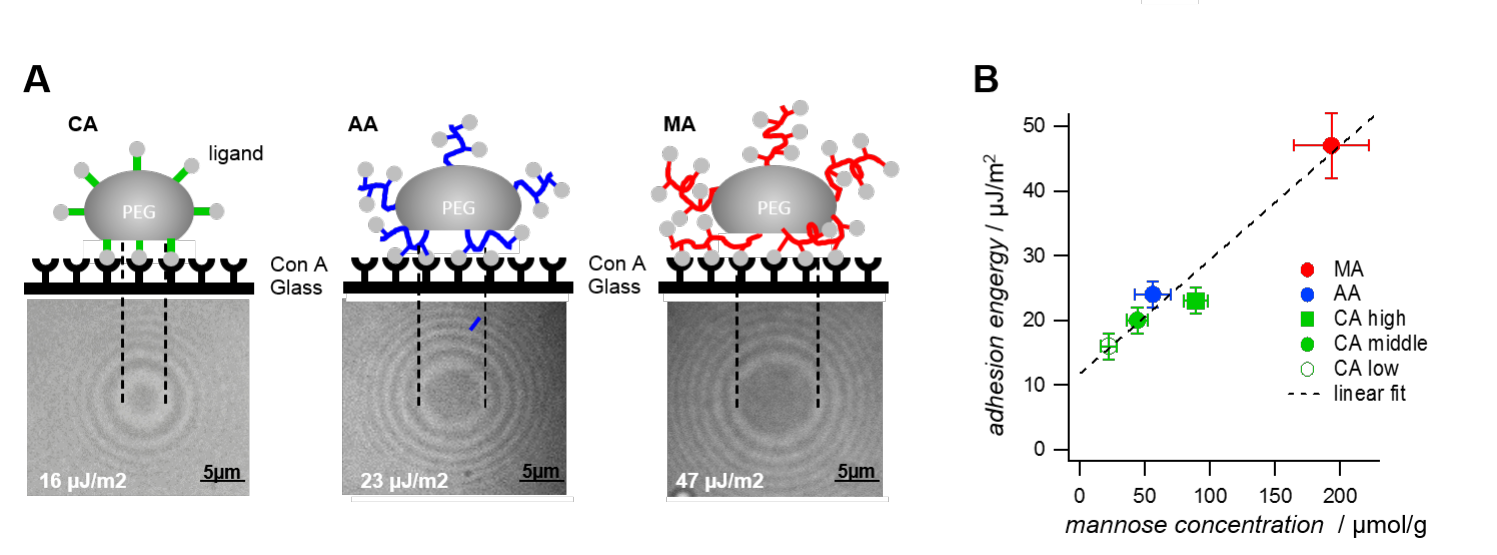
A) Increased mannose densities as schematically shown lead to increased contact areas. For the PEG-CA particles only one mannose unit is attached to the particles per CA graft, whereas for PEG-MA and PEG-AA polyacid chains are present on the surface. B) Plot of the adhesion energy vs mannose concentrations for SCPs with three different grafting types and three different PEG-CA SCPs with varying density of grafting units PEG-CA_low_ PEG-CA_middle_ PEG-CA_high_ (Table 2). The linear fit (*R*^2^ = 0.75) suggests that mannose units have the same affinity regardless of grafting type. Intersection with the y-axis shows indicates the unspecific adhesion energies of unmodified SCPs (data not shown).

With regard to the SCP adhesion assay we observed the largest contact in case of PEG-MA SCPs due to the high degree of functionalization, whereas PEG-AA SCPs showed similar adhesion energies to PEG-CA SCPs of similar degree of functionalization (Figure 4A). This could be explained by the fact that PEG-AA and PEG-CA SCPs exhibit a similar density of mannose units. Importantly, when comparing the mannose density on the different SCPs with the resulting adhesion energy we find a simple linear relation (Figure 4B). This suggests that the overall specific interaction of SCP-bound mannose and the ConA layer is simply directly proportional to the density of mannose units per graft. In other words, the affinity of the individual mannose units is the same between AA, CA and MA-SCPs and does not depend on the grafting characteristics, i.e., the length of grafts and the number of attached mannose units. This is an important result, because a potentially multivalent arrangement of mannose on AA and CA grafting units could lead to chelate- or subsite binding at the ConA receptor enhancing the affinity of individual mannose units. In contrast to other work on similar multivalent scaffolds like oligomers, dendrimers or nanoparticles [20–22] where such chelate- and subsite-binding modes were discussed, our SCP-assay did not indicate enhancement of affinity due to multivalent binding modes for any grafting type. This is surprising as for all SCP systems the density of mannoses is large enough to bind to multiple ConA binding sites: For example, in case of the PEG-MA the density of mannose was ≈200 µmol/g, which translates to ≈4 grafted mannose units per PEG chain (average MW 8000 kDa). Considering the hydrodynamic radius of a PEG 8000 chains of ≈10 nm [8] this would mean that the spacing of mannose units was on the order of 2 nm and therefore sufficiently high for potential multivalent binding to the binding pockets of ConA (separated by ≈6.5 nm). The absence of a multivalency effect could be explained by the large dissociation rates of mannose–ConA complexes prohibiting chelate or sub-site binding. This is caused by the generally low affinity between sugars and receptors and possibly also by the high flexibility of the polymeric mannose linkers [9]. High molecular flexibility causes a high degree of conformational entropy that negatively affects complex formation between ligands and receptors [23]. Also the design of the binding assay could lead to different conclusions on multivalency effects. In typical inhibition/competition affinity assays steric shielding is the main contributor to the observed multivalency effect [24–25] in particular for large polymeric scaffolds. In direct binding assays, as conducted here, steric shielding is not detected, which could explain the different outcome in terms of binding affinity per mannose unit in comparison to studies using inhibition/competition for binding affinity characterization [20–22].

## Conclusion

In this work, we successfully grafted three different carboxylic acid monomers (methacrylic acid, acrylic acid and crotonic acid) on PEG-based SCPs. Methacrylic acid grafts on PEG microparticles result in long poly(methacrylic acid) chains, acrylic acid grafts in shorter oligo(acrylic acid) chains, and crotonic acid grafts in the form of single crotonic acid molecules. Thus, the differently functionalized SCPs vary in both, in their degree of functionalization as well as the multivalent presentation of functional groups (oligo/polymer chains vs single functional groups). Further functionalization of the SCPs with mannose ligands gives a series of sugar-functionalized SCPs with varying degree of functionalization and variation of ligand presentation. The sugar SCPs were then applied in the previously developed SCP-RICM adhesion assay which can be considered as model systems for the interaction of a cell glycocalyx with a protein receptor surface.

The results show that a high mannose concentration generally leads to increased adhesion energies. Although the mannose density was in principle sufficient to form multivalent binding with ConA receptors for all SCP systems, we did not observe an enhancement of binding affinity per mannose unit when increasing the mannose concentration on the particles. This suggests that the surface interactions between mannose and ConA did not lead to multivalent interactions in the sense of a chelate- or subside binding complex. This could be caused by the flexible arrangement of mannose units on the polymer scaffold or insufficient density of the binding partners. In future work we will therefore further increase the density of sugar units, which would ensure closer mimics of the highly dense presentation of ligands within the glycocalyx. Such studies could reveal the optimal ligand surface density and spacing in order to maximize receptor adhesion and selectivity.

## Experimental

### Materials

Benzophenone was purchased from Acros Organics, benzotriazole-1-yloxytripyrrolidinophosphonium hexafluorophosphate (PyBOP) and 1-hydroxybenzotriazole (HOBt) from IRIS. All other chemicals were purchased from Sigma-Aldrich.

### General procedure for the synthesis of carboxylated PEG SCPs

PEG microparticles were synthesized by precipitation polymerization in a similar manner as described in ref. [9]. Briefly, PEG-diacrylamide (*M*_n_ = 8000 Da) [26] (50 mg, 6.3 µmol) was dispersed in a 1 M sodium sulfate solution (10 mL). The UV photoinitiator Irgacure 2959 was added at a concentration of (1 mg, 4.5 µmol) to the dispersion and vigorously shaken and photopolymerized with UV light. Water was exchanged by ethanol and benzophenone (250 mg, 1.4 mmol) and an unsaturated carboxylic acid (acrylic acid (1.2 mL, 17.7 mmol), methacrylic acid (1.5 mL, 17.7 mmol) or crotonic acid (1.5 g, 17.7 mmol) were added and the mixture was flushed with argon for 30 s and irradiated with UV light for 900 s. The microparticles were washed with ethanol 3 times and stored in ethanol. The resulting particles were 20–100 µm in diameter [15].

### Quantification of carboxylic acid in PEG-SCPs

The carboxylic acids in the PEG-SCPs were quantified by titration with TBO, zeta potential measurements and IR spectroscopy. The TBO measurement was conducted as follows. 1.5 mL of PEG-SCP dispersion were centrifuged and a solution of 0.5 mM TBO solution (pH 10.3) was added to the pellet and incubated for 5 h. After several washing steps with sodium hydroxide solution (pH 10.3) the dispersion was centrifuged/washed in 1:1 acetic acid/water mixture collecting defined volumes of the supernatant. The amount of hydrogel was determined gravimetrically after drying the SCPs and the amount of carboxylic groups was determined photometrically using the released TBO in the supernatant.

For zeta potential measurements 1 mL of carboxylic acid-functionalized PEG-SCPs were injected into a Malvern DTS1060 disposable folded capillary cell. The Zeta-potential was characterized with a Malvern Instruments Nano Series ZS ZEN3500 Zetasizer at 25 °C in Milli-Q water (Type I Water). Each sample was tested 3 times and the averaged value was recorded as the the Zeta-potential of this sample.

### General procedure for the synthesis of PEG-Man microparticles

In a similar manner as described in [9] ethanol was exchanged with DMF through several washing steps and the carboxylate-functionalized microparticles (0.03 g) were left in 10 mL of DMF. PyBOP (0.728 g, 1.40 mmol), HOBt (0.097 g, 0.70 mmol) and triethylamine (195 µL, 1.40 mmol) were added to activate the carboxylic groups. This suspension was shaken for 15 min at rt, then aminoethyl-linked acetyl protected mannose [8,8] (0.050 g, 0.12 mmol) was added. The mixture was allowed to react for 3 h at rt. The microparticles were centrifuged and washed 3 times with DMF and 3 times with methanol. Sodium methoxide (0.004 g, 0.08 mmol) were added and reacted for 1 h at rt for deprotection. Then, the microparticles were centrifuged at 5000 rpm for 10 min and washed 3 times with methanol and 3 times with pure water.

### Determination of the SCPs elastic modulus

To calculate the adhesion energies of the SCPs, the elastic modulus of the particle is required (see Equation 1). AFM force spectroscopy with a NanoWizard 3 system (JPK instruments AG, Berlin, Germany) was performed to determine the elastic modulus of the microparticles. As AFM probe a glass bead with a diameter of 5.1 µm was glued with an epoxy glue onto a tipless, non-coated cantilever (spring constant 0.32 N/m; CSC12, NanoAndMore GmbH). Several force curves were recorded from different particles and analyzed with an appropriate contact model developed by Glaubitz et al. [27]. The elastic moduli of PEG-SCPs were 32 ± 5 kPa and showed no systematic variation with regard to grafting type or degree of mannose functionalization.

### Immobilization of ConA to glass surfaces

ConA was bound to coverslips as previously described [8]. Briefly, coverslips (Ø 24 mm, ≈0.17 mm thickness) were used as glass surface (Thermo scientific, Germany) and cleaned prior to use by washing with isopropanol and piranha solution (96% H_2_SO_4_ and 30% H_2_O_2_, 3:1). The coverslips were rinsed with ultra-pure water and dried in a nitrogen stream. Amine surfaces were prepared via chemical vapor deposition of 3-aminopropyltriethoxysilane (APTES) (50 µL) on a freshly cleaned coverslip were placed in a desiccator and vacuum was applied for 1 min. The desiccator was sealed and the coverslips were left for 1 h to react with the vapor. The coverslips were rinsed with Milli-Q water and dried with nitrogen. Then the coverslips were placed in PBS buffer pH 7.4 containing 2.5% glutaraldehyde for 30 min followed by washing with Milli-Q water and drying. ConA (0.2 mg mL^−1^) in PBS buffer pH 7.4 was placed on the aldehyde-functionalized surfaces for 1 h [19]. and prior to the measurements washed with lectin binding buffer (10 mM HEPES pH 6, 50 mM NaCl, 1 mM MnCl_2_, 1 mM CaCl_2_).

### Reflection interference contrast microscopy (RICM)

RICM on an inverted microscope (Olympus IX73, Germany) was used to obtain the contact area between the microparticles and a hard glass surface. For illumination an Hg-vapor arc lamp was used with a green monochromator (546 nm). A Zeiss Antiflex 63× NO 1.25 oil-immersion objective, additional polarizers to avoid internal reflections and a Zeiss AxiocamHRm camera were used to image the RICM patterns. To conduct the JKR measurements of the adhesion energies, both the contact radius and the particle radius were measured. Image processing and data analysis were done using the image analysis software ImageJ (public domain NIH) and the mathematical software OriginPro (OriginLab, USA). 1 mL of lectin binding buffer pH 6 was added to the ConA-functionalized surface and PEG-Man SCPs were spread into the solution. The particles were sedimented and the contact radius and the particle radius were measured. Inhibition of the interaction was done by adding of α-methyl-D-mannose (300 µL, 1 mg mL^−1^) in lectin binding buffer to the suspension and well mixed so that all bound particles were detached from the surface.

## Supporting Information

File 1 Additional experimental and analytical data.
