# Supplementary material for: Probing multivalency in ligand–receptor-mediated adhesion of soft, biomimetic interfaces
Source: Beilstein J Org Chem. 2015 May 12;11:720–9. doi: 10.3762/bjoc.11.82 (PMC4464160; doi:10.3762/bjoc.11.82)
Supplement: File 1 — Additional experimental and analytical data. [file Beilstein_J_Org_Chem-11-720-s001.pdf]

Supporting Information

for

Probing multivalency in ligand–receptor-mediated adhesion of soft, biomimetic interfaces

Stephan Schmidt[1], Hanqing Wang[2,3], Daniel Pussak[3], Simone Mosca[3], and Laura Hartmann[*,§,2,3]

Address: [1]Universität Leipzig, Institut für Biochemie, Johannisalle 21–23, D-04103 Leipzig, Germany, [2]Max Planck Institute of Colloids and Interfaces, Research Campus Golm, 14424 Potsdam, Germany and [3]Heinrich-Heine-Universität Düsseldorf, Institut für Organische und Makromolekulare Chemie, Universitätsstr. 1, 40225 Düsseldorf, Germany

Email: Laura Hartmann - laura.hartmann@hhu.de

*Corresponding author

[§]Tel. ++49 211 81-10360, Fax: ++49 211 81-15840

Additional experimental and analytical data

Supporting Information S1: Zeta Potential Measurements

Table S1: Zetapotential measurements (in mV) on PEG-AA, PEG MA, PEG-CA SCPs.

Sample No.	AA	MA	CA
1	-36.0	-50.0	-27.9
2	-29.7	-51.9	-26.4
3	-31.3	-50.1	-24.5
Average	-32.3	-50.7	-26.3
Std. deviation	3.3	1.1	1.7

Supporting Information S2: Raman Microscopy indicates homogeneous distribution of carboxylic coupling groups in the PEG-SCPs

Raman spectrum of PEG-AA. The SCP is divided in three parts: core (blue), middle (green) and surface (red). The spectra were normalized to the CH band at 1480 cm^{-1}. The carbonyl functionalities are homogenious distributed over the whole SCP (1720 1/cm).

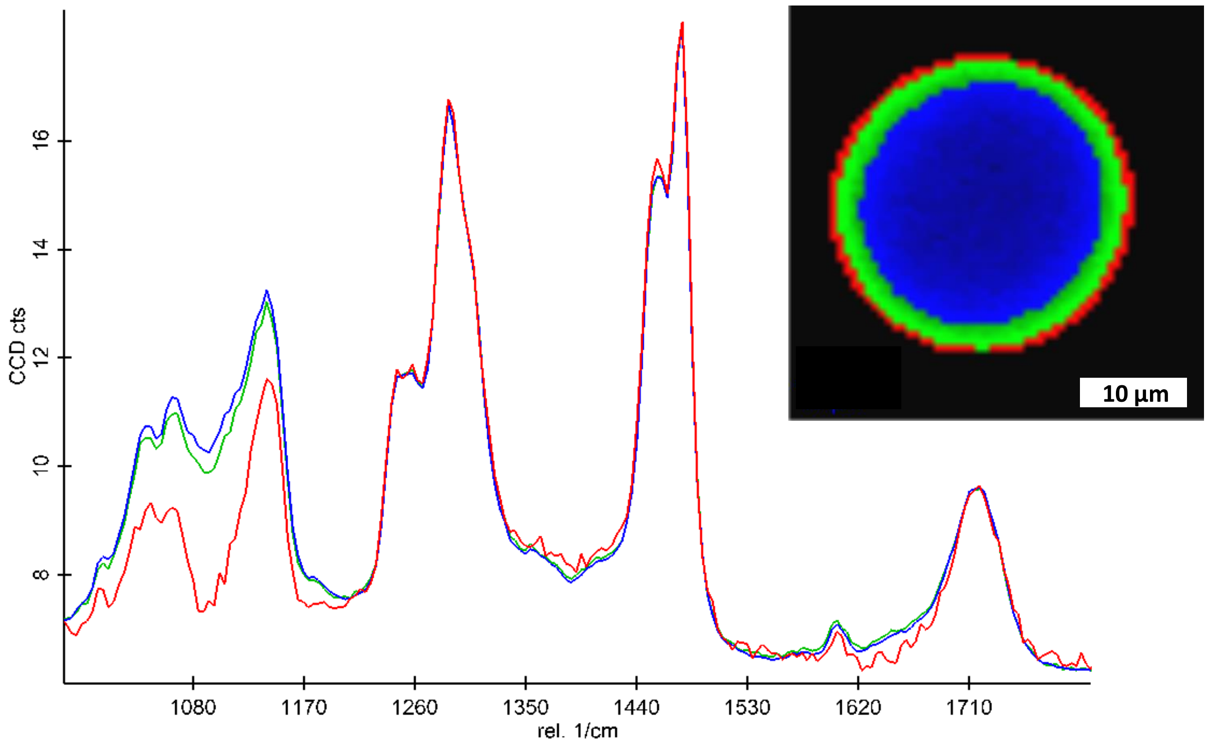

Supporting Information S3: Complete presentation of numerical values, density of carboxylic and mannose groups, and adhesionenergies

Results of the functionalization of the carboxylate functionalized particles with mannose and its corresponding adhesion energy per square meter values.

Sample	Concentration of carboxyl groups (µmol/g)	Concentration of unreacted carboxyl groups (µmol/g)	Concentration of mannose groups (µmol/g)	Adhesion energy per square meter (µJ/m^2)
PEG-CA_{low}	36 ± 2	14 ± 4	22 ± 6	16 ± 2
PEG-CA_{middle}	57 ± 5	13 ± 3	44 ± 8	20 ± 2
PEG-CA_{high}	97 ± 7	8 ± 2	89 ± 9	23 ± 2
PEG-AA	117 ± 9	61 ± 5	56 ± 14	24 ± 2
PEG-MA	259 ± 24	66 ± 5	193 ± 29	47 ± 5

Supporting information S4: JKR plots for surface energy calculation

SCP-RICM adhesion measurements of PEG-MAA (●), PEG-AA (■), PEG-CAhigh (▲), PEG-CAmiddle (►) and PEG-CAlow (▼) against ConA functionalized glass surfaces.

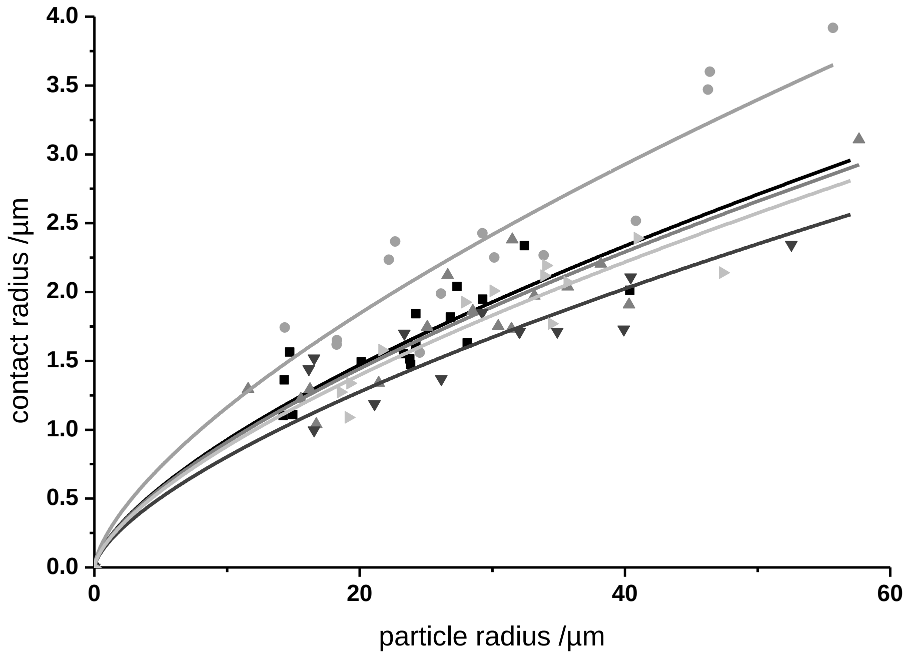

Supporting information S5: Reaction scheme of mannose coupling to SCPs

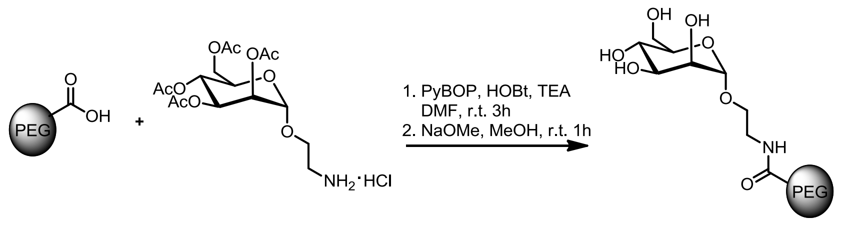

Supporting information S6: Reflection Interference Contrast Microscopy (RICM) measurements

Setup

RICM on an inverted microscope (Olympus IX73) was used to obtain the contact area between the microparticles and a hard glass surface. For illumination a monochromatic (530 nm) collimated LED (Thorlabs, Germany, M530L2-C1) was used. An Olympus 60 x NA 1.35 oil-immersion objective (UPLSAPO60XO/1,35 U Plan S Apo), additional polarizers and a quarter waveplate (Thorlabs, germany) to avoid internal reflections and a monochrome CMOS camera (UI-3360CP-M-GL, IDS Germany) were used to image the RICM patterns. To conduct the JKR measurements, both the contact radius (in RICM mode) and the particle radius (in transmission mode) were measured. Image acquisition was done using µManager (v1.4.16), data analysis was done using the image analysis software Image-J (v1.48) and the mathematical software IgorPro (v6.38, Wavemetrics, USA).

Determination of the contact radius

RICM was used to measure the contact radius formed by the SCPs resting on the polymer surface (Figure S6a). Polarized light waves reflected from the upper glass surface (I_1) and the surface of the bead (I_2) interact to create an interference image. The intensity at a given position in the image depends on the separation $h(x)$ between the two surfaces: $I(x) = I_1 + I_2 + 2\cdot\mathrm{sqrt}(I_1 \cdot I_2)\cos[2k\cdot h(x) + \pi]$, where $k = 2\pi n/\lambda$, and n and λ are the index of refraction of water and the wavelength of the monochromatic light, respectively. In order to detect the interference pattern, stray light was reduced by an ‘antiflex’ technique. This is accomplished by crossed polarizer and analyzer filter with a $\lambda/4$-plate placed between the objective lens and the analyzer [1].

Practical note: Although it is generally recommended to use the antiflex optics with polarization methods to avoid stray light generated in the microscope, we observed only little improvement in image contrast when using the antiflex setup. RICM images could be readily taken without polarizer, analyzer and quarter wave plate. This is possibly due to the rigorous use of antireflective lenses in the microscope and Thorlabs components.

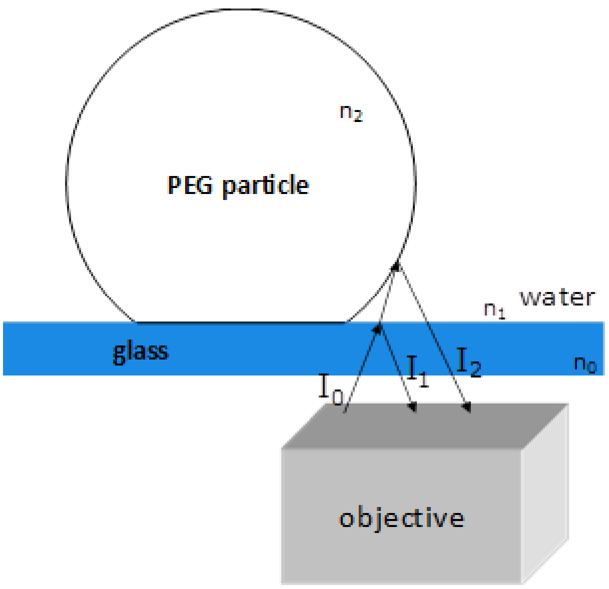

Figure S6a: Schematic drawing of the RICM principle.

Correction Factors

For analysis of the intensity distribution correction factors must be determined for finite aperture and geometry effects. To obtain the correction factors, we imaged hard, non-deformable glass beads on a glass surface in RICM mode with a known size. We recorded 5 glass beads with a diameter in the range of 10-20 µm and extracted the intensity profile. Using the profiles, we reconstructed the shape of the beads and compared it to the known spherical shapes of the glass beads (glass bead radius R measured by light microscope), and determined the correction factors, see Pussak et al. [2].

Contact radius determination

To determine the contact radius *a* of the SCP on the polymer surface we reconstructed the height profile of the particles from the RICM images (see Figure S6b). This was done by determining the lateral *x*(*i*) positions of the *i*-th minima and maxima by a self-written IgorPro procedure (Wavemetrics, USA). Next, the vertical position *y*(*i*) of the maxima and minima were determined by

$$y(i) = \frac{i\lambda}{4n} + c_i ,$$

where *n* is the refractive index and λ the wavelength. The height profile was then reconstructed by plotting *y*(*i*) vs *x*(*i*) and fitting the data by a circle equation representing the assumed shape of the SCP:

$$y(x) = y_0 + \sqrt{R^2 - x^2} .$$

where *R* is the independently measured SCP radius and y_0 the vertical shift of the SCP center due to flattening of the SCP upon adhesion. The fit with y_0 as the only free fit parameter intersects with the x-axis and gives the contact radius *a*.

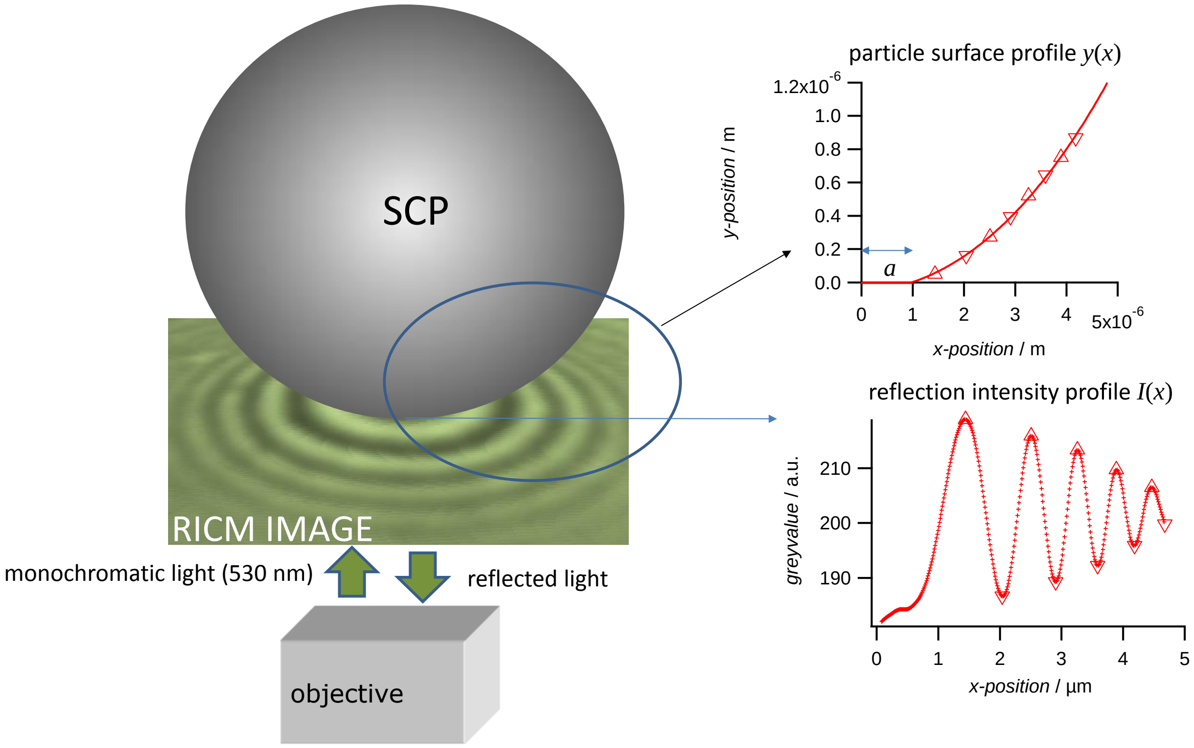

Figure S6b Left: schematic representation of the measurement setup. Bottom right: actual intensity profile of an adherent SCP showing 5 minima and 5 maxima. Top right: reconstructed surface profile of the SCP and the contact radius *a* at the intersection of the profile at *y* = 0.

Supporting References:

1. Limozin, L.; Sengupta, K.,*ChemPhysChem* **2009,** *10* (16), 2752-2768.
2. Pussak, D.; Ponader, D.; Mosca, S.; Ruiz, S. V.; Hartmann, L.; Schmidt, S.,*Angew. Chem. Int. Ed.* **2013,** *52* (23), 6084-6087.
